# A microfluidic-based filtration system to enrich for bone marrow disseminated tumor cells from breast cancer patients

**DOI:** 10.1371/journal.pone.0246139

**Published:** 2021-05-14

**Authors:** Sreeraj G. Pillai, Chidananda M. Siddappa, Cynthia Ma, Jackie Snider, Madhurima Kaushal, Mark A. Watson, Rebecca Aft

**Affiliations:** 1 Dept. of Surgery, Washington University School of Medicine, St. Louis, MO, United States of America; 2 Dept. of Medicine, Washington University School of Medicine, St. Louis, MO, United States of America; 3 Dept. of Pathology and Immunology, Washington University School of Medicine, St. Louis, MO, United States of America; 4 Institute of Informatics, Washington University School of Medicine, St Louis, MO, United States of America; 5 John Cochran Veterans Administration Hospital, St. Louis, MO, United States of America; Hunter College, UNITED STATES

## Abstract

Disseminated tumors cells (**DTCs**) present in the bone marrow (**BM**) are believed to be the progenitors of distant metastatic spread, a major cause of mortality in breast cancer patients. To better understand the behavior and therapeutic vulnerabilities of these rare cell populations, unbiased methods for selective cell enrichment are required. In this study, we have evaluated a microfluidic-based filtration system (Parsortix^R^, Angle PLC), previously demonstrated for use in circulating tumor cell (**CTC**) capture, to capture BM DTCs. Performance using BM samples was also compared directly to enrichment of CTCs in the peripheral blood (**PB**) from both metastatic and non-metastatic breast cancer patients. Although the non-specific capture of BM immune cells was significant, the device could routinely achieve significant cytoreduction of BM and PB WBCs and at least 1,000-fold enrichment of DTCs, based on labeled tumor cell spike-in experiments. Detection of previously characterized DTC-associated gene expression biomarkers was greatly enhanced by the enrichment method, as demonstrated by droplet digital PCR assay. Cells eluted from the device were viable and suitable for single cell RNA sequencing experiments. DTCs in enriched BM samples comprised up to 5% of the total cell population, allowing for effective single cell and population-based transcriptional profiling of these rare cells. Use of the Parsortix instrument will be an effective approach to enrich for rare BM DTCs in order to better understand their diverse molecular phenotypes and develop approaches to eradicate these cells to prevent distant disease development in breast cancer patients.

## Introduction

Distant metastases development is a significant cause of mortality in breast cancer (**BC**) patients. Disseminated tumor cells (**DTCs**) are believed to be the precursors to metastatic disease after the primary tumor is removed [1–3]. Enormous effort has been devoted to identifying and molecularly characterizing these rare cells for therapeutic targeting before they progress to overt metastatic foci. The most readily accessible DTCs for study in early-stage breast cancer patients are those isolated from the bone marrow (**BM**). BM DTCs are associated with recurrent disease development and poor prognosis [2, 4] even years after initial diagnosis [5]. Patients with detectable BM DTCs after chemotherapy are at very high risk of recurrence, indicating that these DTCs may have high metastatic potential [6].

Studying DTCs has several advantages over circulating tumor DNA (**ctDNA**) or circulating tumor cells (**CTCs**), both of which had been associated with disease progression [7–9]. First, DTCs are 10–250 fold more abundant than CTCs in early stage BC patients, thus more amenable to molecular and cellular investigation [10, 11]. Second, in BC patients, DTCs appear to be more closely associated with clinical outcome and disease progression compared to ctDNA and CTCs [12, 13]. Efforts to isolate, identify, and molecularly characterize DTCs from patient BM specimens have been hindered by the heterogeneity of cells and the cellular complexity of BM. Phenotypic transitioning of DTCs as they adapt to changing micro-environments has resulted in a lack of uniform molecular markers that predict metastatic potential [14].

Multiple techniques have been developed to enrich for rare cells such as DTCs and CTCs for subsequent molecular analysis [15]. These methods have been based on the physical and/or molecular properties of the cells. Antibody-based techniques have been employed focusing on specific surface antigens, such as EpCAM, to positively select target cells or by negative selection through elimination of contaminating leukocytes by targeting leukocyte specific antigens, such as CD45 (reviewed in [16]). However, DTCs may escape these affinity binding methods due to their heterogeneity and loss of epithelial antigens [17, 18]. Other enrichment platforms have been developed for rare cells based on physical properties such as cell size, density, or decreased deformity (reviewed in [19]). Filtration methods exploit size disparities between cancer cells and normal hematopoietic cells, which allows antigen-independent collection and currently, several such systems are available (reviewed in [20–22]). We have previously optimized a filtration system for DTC retrieval from BM [23].

To assess a system for easy retrieval of viable rare cancer cells, we evaluated the use of an epitope-independent microfluidic cell separation technology (Parsortix) for isolating DTCs from BM and performing downstream analysis. This technology is based on the size and deformability of cells [24–26] and allows the retrieval of viable cancer cells. This system has been used in enrichment of CTCs from blood samples of cancer patients [24, 27–30].

## Methods

### Ethics statement/patient population

This study was approved by the Institutional Review Board at Washington University in St. Louis. All patients were recruited from the Siteman Cancer Center and Washington University Medical Center. Enrolled patients had BM and blood collected between September 2017 and June 2018. Clinical stage II/III breast cancer patients, age range 40–65, of all breast cancer subtypes eligible for neoadjuvant chemotherapy were enrolled into a prospective tissue collection study. Blood from patients with metastatic estrogen receptor positive breast cancer was collected as part of an IRB approved clinical trial entitled “a phase II clinical trial assessing the safety of an alternative dosing schedule of palbociclib in metastatic hormone receptor positive BC (NCT03007979)”. BM was collected from healthy female patients aged 50–65 who were undergoing hip replacement procedure. Written Informed consent was obtained from all patients and healthy volunteers who participated in this study.

### Bone marrow and blood collection

BM and blood were collected from patients with clinical stage II/III breast cancer in the operating room during surgical procedures prior to the initiation of treatment. For patients with metastatic breast cancer, blood was collected in the clinic. 7–10 ml of blood was collected into K_2_-EDTA vacutainer tubes. For BM aspirates, 10ml of aspirate was collected into non-heparinized syringes from normal volunteer posterior iliac crest during orthopedic procedures or from the anterior iliac crest from breast cancer patients, as previously described [23, 31]. Collected BM was placed directly into K_2_-EDTA tubes from a needleless syringe to avoid cell shearing.

### Specimen processing and spiking cancer cells

**Fig 1:**
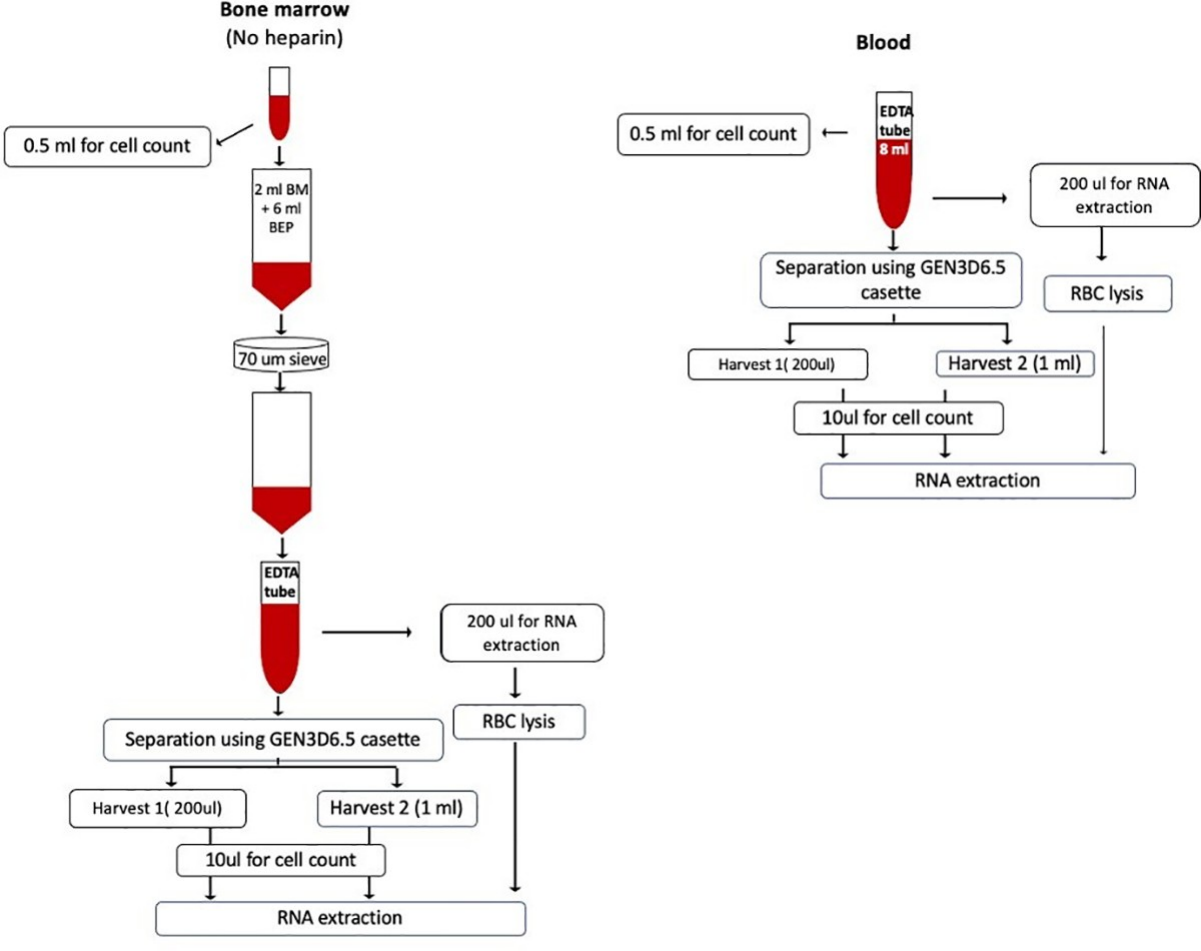
CTC/DTC enrichment workflow using Parsortix system.

Blood and BM nucleated cell counts were determined prior to processing using a Vision CBA cellometer (Nexcelom Biosciences). Workflow for specimen processing is shown schematically in **Fig 1**. Briefly, 8–10 ml of blood in EDTA tubes was used directly for microfluidic separation. For BM samples, 2 ml BM was diluted to 8 ml in BEP buffer (0.5% BSA and 2mM EDTA in 1X PBS) in EDTA tubes to maintain equivalent nucleated cell counts, viscosity and specimen volume as blood samples and to limit the input number of nucleated cells to <50 million total for optimal cell isolation, as recommended by the manufacturer. Diluted BM specimens were subsequently passed through a 70u cell strainer (Fisher Scientific) to remove particulates prior to microfluidic separation.

For cell spiking experiments, SKBR3 BC cells were fluorescently labelled using Celltrace CFSE cell proliferation kit (Life Technologies Corporation, Carlsbad USA) following the manufacturer’s protocol. Briefly, cells were trypsinized and pelleted at 250XG for 5 minutes, washed with PBS and re-pelleted. Cell pellets were resuspended in PBS at a concentration of 1X 10^6^ cells per ml. 1 ml of this cell suspension was mixed with Celltrace CFSE solution to a working concentration of 5uM. The cells were incubated at 37°C for 5 minutes and the reaction terminated with 5 ml of complete growth media (McCoy’s 5A with 10% FBS). Cells were then pelleted and resuspended in PBS. Labelled cells were counted using a hemocytometer and a known number of cells added to blood or BM samples.

### Cell capture using the Parsortix system

Isolation of cancer cells from blood and BM was conducted using the Parsortix PR1 automated microfluidic system (Angle North America, Philadelphia, USA). Details on the mechanism of this system has been described [25]. Disposable cassettes with a gap size 6.5 um (GEN3D6.5) were used unless otherwise specified. Briefly, the separation cassette was primed using alcohol and PBS according to the manufacturer’s recommendation. 8 ml of whole blood or diluted BM was loaded into the device. Cells captured in the cassette were harvested by reversing the flow direction and capturing cells in 200ul PBS. An optional second harvest with 1 ml PBS was performed according to the manufacturer’s recommended protocol. The cells from the initial 200ul harvest (**H1**) and second 1 ml harvest (**H2**) were collected separately. The number of cells eluted in each harvest were counted using a hemocytometer. Those samples with H1 cell counts outside ± 3X standard deviations of the average of the entire sample set, were considered outliers and excluded from further calculations of fold reduction in nucleated cells and average harvest. For tumor cell spiking experiments, fluorescently labelled SKBR3 cells in H1 and H2 as well as those retained in the cassette after the completion of the harvest cycles were counted under a fluorescent microscope. *Capture rate* was defined as the number of cancer cells trapped in the cassette divided by the total number of spiked cancer cells times 100. *Harvest rate* was defined as the number of cancer cells retrieved from the cassette divided by the number of cancer cells trapped in the cassette times 100. *Recovery* was defined the number of cancer cells retrieved from the cassette divided by the total number of spiked cancer cells times 100. *Purity* was calculated as the ratio of retrieved cancer cells to total cells recovered. *Fold enrichment* was calculated as the total number of retrieved cells divided number of total cells loaded into the cassette. *Enrichment of cancer cells* was calculated as the ratio of fluorescent cancer cell concentration in the input sample to the concentration of the harvest harvested cancer cells after parsortix separation.

### RNA purification and mRNA expression analysis

Total RNA was isolated from approximately 1x10^6^ BM or peripheral blood nucleated cells, prior to filtration, as well as the entire cell pellets from the H1 and H2 eluate of the Parsortix chamber using Nucleospin RNA Plus XS columns (Takara), following the manufacturer’s protocols. Eluted RNAs were quantified using a Nanodrop spectrophotometer or Qubit fluorometer and when yields were sufficient, qualitatively assessed by Agilent bioanalyzer. Either 100 ng of total RNA or one-half of the entire RNA yield (when RNA yields from H1 eluates were too low to quantify) was used to generate corresponding cDNA using the iScript cDNA synthesis kit (Biorad). After cDNA synthesis, 50% of the reaction was then used for duplicate targeted, primer-based pre-amplification using SSO Advanced pre-amplification mix (Biorad) and 10 amplification cycles. Each pre-amplification reaction was then subjected to digital droplet PCR (**DDPCR**) amplification using gene-specific primer / probes and the QX200 droplet generator / reader system (Biorad). Primer / probe details used for gene-specific amplification of DTC-associated transcripts (*EPCAM*, *ERBB2*, *PDL-1*, *PDGFRB*, *STEAP1*, *TWIST1*, *WNT5A*) are provided in **S1 Table** [32]. Droplet counts from duplicate reactions were averaged and normalized to counts from GAPDH primer / probes in each sample to account for differences in input cell number and RNA yield and quality between pre- and post-filtration samples, and between patients.

### Single cell RNA sequencing (scRNAseq) analysis

For single cell RNA analyses (scRNAseq), harvested samples from the H1 eluate with >1.5 x 10^4^ viable cells were used for library generation. Cell suspensions were provided to the Washington University McDonnell Genome Institute for creation of single cell 3’ libraries using the 10X Genomics Chromium system. cDNA was prepared using the standard 10X Genomics library preparation protocol using GEM generation and barcoding, followed by the GEM-RT reaction and bead cleanup steps. Purified cDNA was amplified for 11–13 cycles before being cleaned up using SPRI select beads. The sample was then run on a Bioanalyzer to determine the cDNA concentration. A Gene Expression library was prepared as recommended by the 10x Genomics Chromium Single Cell 3’ Reagent Kits (v3 Chemistry) user guide with appropriate modifications to the PCR cycles based on the calculated cDNA concentration. For sample preparation on the 10x Genomics platform, the Chromium Single Cell 3’ GEM, Library and Gel Bead Kit v3 (PN-1000075), Chromium Single Cell B Chip Kit (PN-1000153), and Chromium i7 multiplex kit, 96rxns (PN-120262) were used. The concentration of the library was accurately determined through qPCR utilizing the KAPA library Quantification Kit according to the manufacturer’s protocol (KAPA Biosystems/Roche) to produce cluster counts appropriate for the Illumina NovaSeq6000 instrument. The library was then sequenced on a NovaSeq6000 S4 Flow Cell using the XP workflow and a 28x8x98 sequencing recipe according to manufacturer protocol. A median sequencing depth of 50,000 reads/cell was targeted for the Gene Expression Library.

As a secondary quality assurance check, libraries that yielded <100 ng of cDNA were rejected to avoid analyzing samples with a low cell viability which may result in low cell / transcript representation or numbers. A total of 6 independent BM DTCs enrichments were submitted for 10X library creation. Of these, three samples resulted in low cDNA yields were not analyzed further. Three samples yielded adequate cDNA and libraries and proceeded to sequencing; one of those samples is described in this report to illustrate feasibility. Cluster (**tSNE**) analysis of single cell expression data sets were performed using output from CellRanger (10X Genomics). Additional cell cluster annotation was created using SingleR package in Bioconductor. Single cell RNA analysis data are available at Gene Expression Omnibus (GEA: GSE162334).

### Statistical analysis

The statistical significance of harvest cell count and harvest to total cell count ratios between different sample groups were determined by t-test using Graphpad software.

Dimensional reduction, clustering, and analysis of single-cell RNA sequencing data was performed using the R package Seurat version 3.2.2. Cells with expression of fewer than 200 or more than 5000 genes were filtered out of the analysis. The data was normalized using "LogNormalize" method of Seurat with a scaling factor of 10000 and variable genes were identified using the Seurat "FindVariableFeatures" method. Principal component analysis (**PCA**) for dimensional reduction was performed using Seurat functions based on the variable genes previously identified. Cell clustering and tSNE visualization were performed using the "FindClusters" and "RunTSNE" functions, respectively. To identify how the expression of the genes of interest changed across the different clusters, a dotplot was created using Seurat function "DotPlot". The size of the dot in the plot corresponds to the percentage of cells expressing the gene in each cluster and the color represents the average expression level.

## Results

### Cytoreduction of nucleated cells from bone marrow and blood

The Parsortix system has been optimized for CTC enrichment from peripheral blood [26]. Because BM is many fold more cellular than peripheral blood and because the size and characteristics of immature immune cells found in the BM differ from those of mature peripheral blood granulocytes and mononuclear cells, we first evaluated whether the Parsortix system could effectively perform background cytoreduction of appropriately diluted BM specimens. We initially tested available microfluidic cassettes with size gaps of 6.5–10 microns. The 6.5 micron gap cassette was optimal for retaining the greatest number of tumor cells with the lowest number of contaminating WBC and was used for further experiments (**S2 Table**). To test the efficacy of the Parsortix system for BM cyto-reduction, 16 BM samples from clinical stage II/III BC patients were processed and the results compared to that of 13 peripheral blood samples from non-metastatic patients, and 39 peripheral blood specimens from metastatic patients. Among the 68 samples processed, 3 (4%) samples,1 BM and 2 from metastatic BC patients, were considered ‘failures’ and excluded from the analysis since cell counts from the H1 elution were over three standard deviations above the average yield for all other samples, despite no obvious differences in input cell number, specimen quality, or patient characteristics. These 3 failures were attributed to mechanical issues with the cartridge or the fluidics system.

**Fig 2:**
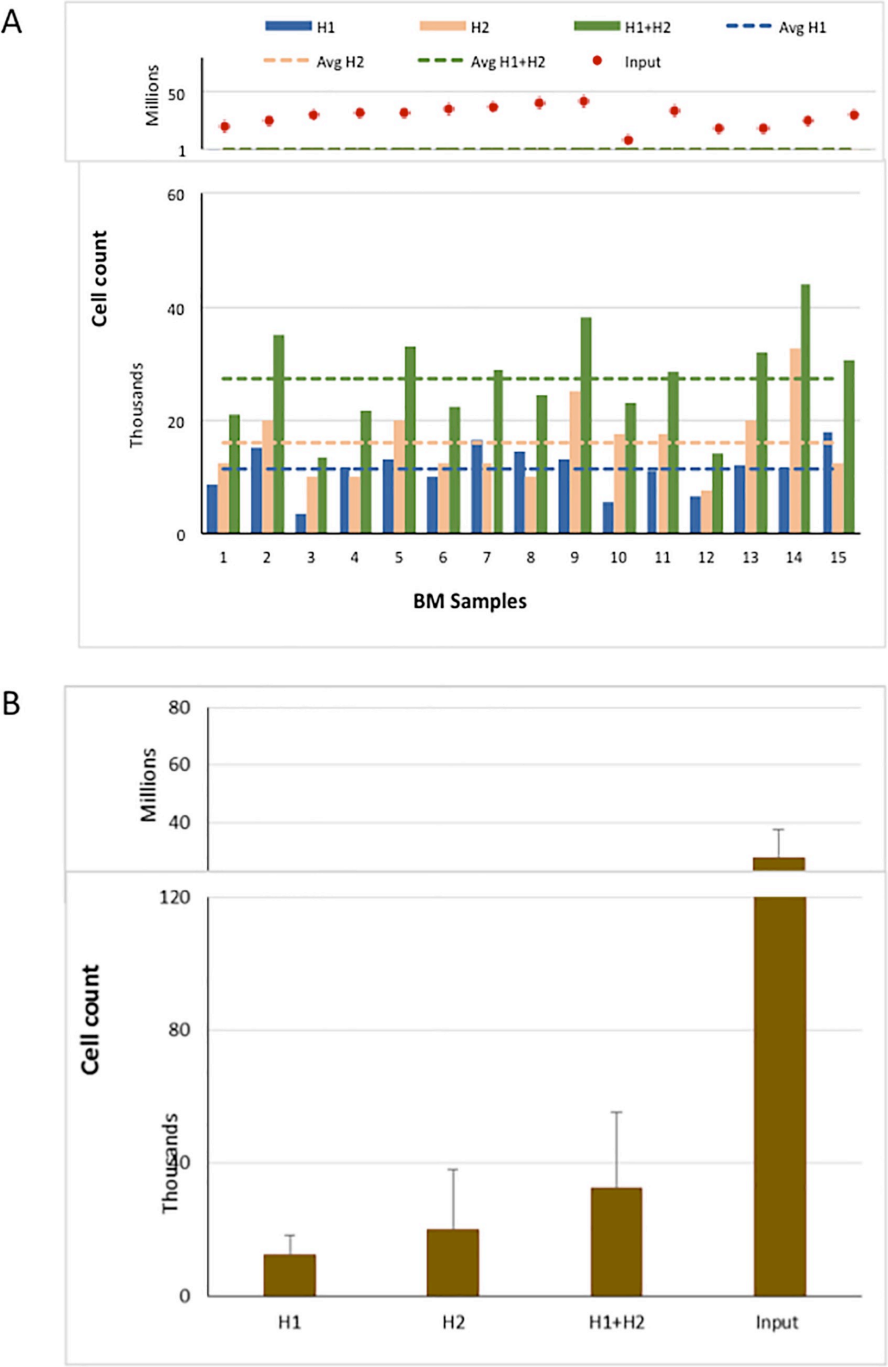
Yield of nucleated BM cells using the Parsortix system.

The extent of cytoreduction of nucleated cells in BC patient BM was calculated by comparing the total number of input cells and the number of nucleated cells harvested in the H1 and H2 eluates, independently. The average input number of nucleated BM cells was 29 million (range 9–43 million) and the average number of nucleated cells in H1 was 11,300 (range 3,500–18,000) for an average of 2,550 fold reduction in nucleated cells (**Fig 2A**). There was no correlation between the number of input cells and nucleated cells in H1 eluate, which has been previously observed for CTCs [28]. In an attempt to increase the recovery of potentially captured tumor cells (see below), a second wash (**H2**) was performed. However, the yield of BM nucleated cells was markedly increased when H1 and H2 eluates were combined, resulting in a two-fold decrease in cyto-reduction efficiency (average 1,050-fold vs 2,550-fold, **Fig 2B**).

Total nucleated cell input and count in harvest fractions of BM specimens (A) and average number of cells in each fractions (B).

**Fig 3:**
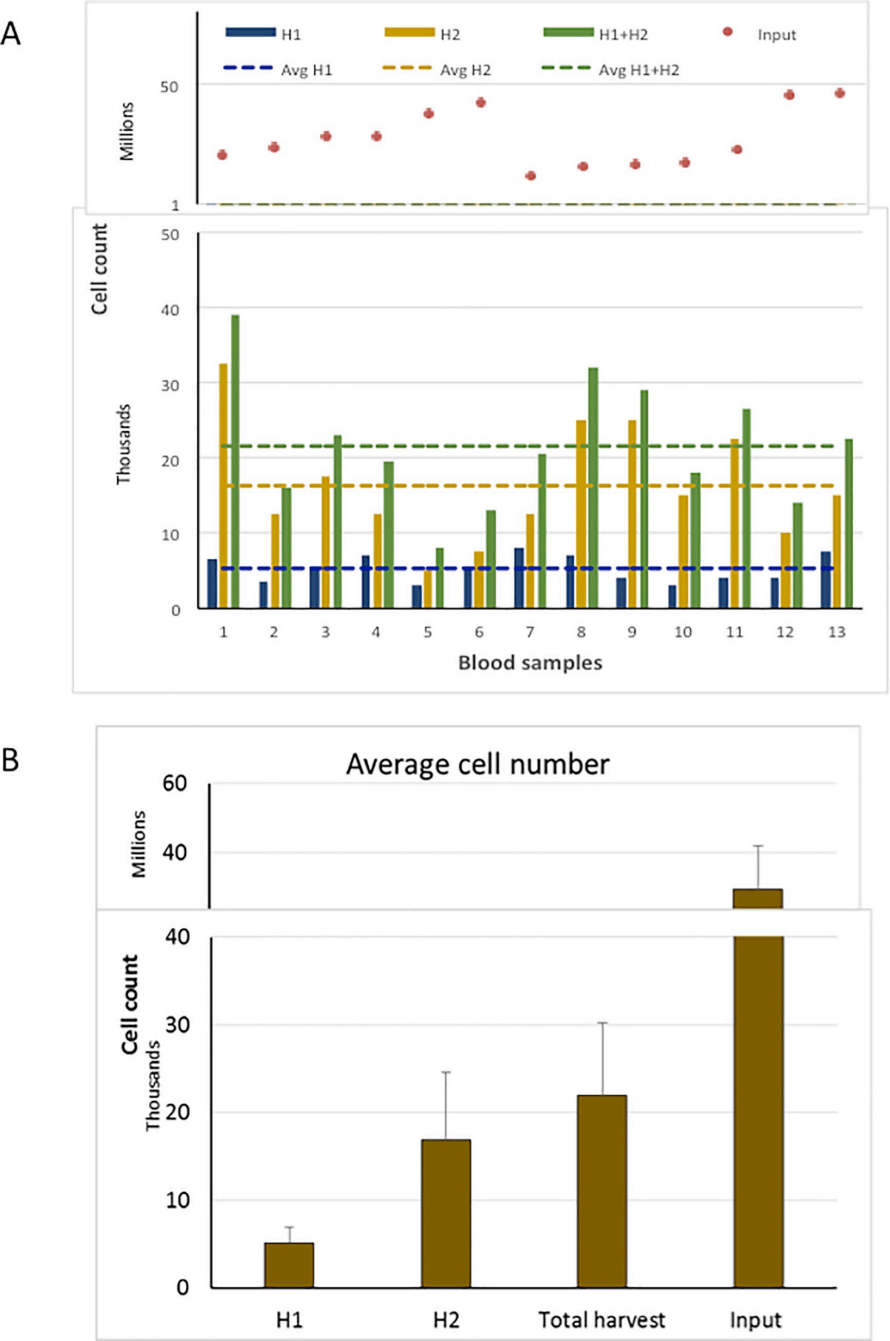
Yield of nucleated blood cells from patients with early stage breast cancer using Parsortix system.

For comparison, we also performed cyto-reduction of peripheral blood samples from both non-metastatic and metastatic BC patients. For early stage breast cancer patients, six of the peripheral blood and BM were collected from the same patient (Samples 1–6 in **Figs 2A** and **3A).** For blood samples from patients with early stage BC, the starting cell number and nucleated cells harvested are shown in **Fig 3A.** The average input number of nucleated blood cells was 28 million (range 12–47 million) and the average number of nucleated cells in the H1 eluate was 5,270 (range 3,500–8,000) for an average fold reduction of 5950 (**Fig 3B**), which is consistent with other reports (**S3 Table**). There was no correlation between the number of input cells and nucleated cells retrieved in H1 eluate for the blood samples, nor was there any correlation of nucleated cell yields between blood and BM from the same patient. There was an increase in the number of nucleated cells when H1 was combined with H2, resulting in a decreased fold reduction of nucleated cells (1300 fold compared to 5950 fold).

Total nucleated cell input and count in harvest fractions of blood specimens (A) and average number of cells in each fractions (B).

In early stage BC patients, a two-fold greater cyto-reduction was observed with blood specimens than observed for BM specimens (5270 versus 11300, blood and BM, respectively) despite similar input cell numbers. Similar results were observed in the 6 patients with both blood and BM analyzed (average yield of nucleated cells 5500 versus10,000 blood and BM, respectively) (**S1 Fig**). The ratio of cell number harvested to total cell input was significantly higher in BM specimens compared to blood samples (.0004, .0002, respectively, p = 0.003), resulting in a decreased fold reduction of nucleated cells in BM compared to blood (2550 versus 5950). Higher retention of nucleated cells in BM samples was anticipated due to the increased cellular complexity of the BM compared to blood which includes larger sized precursor cells as compared to mature lymphocytes and granulocytes found in the peripheral circulation.

**Fig 4:**
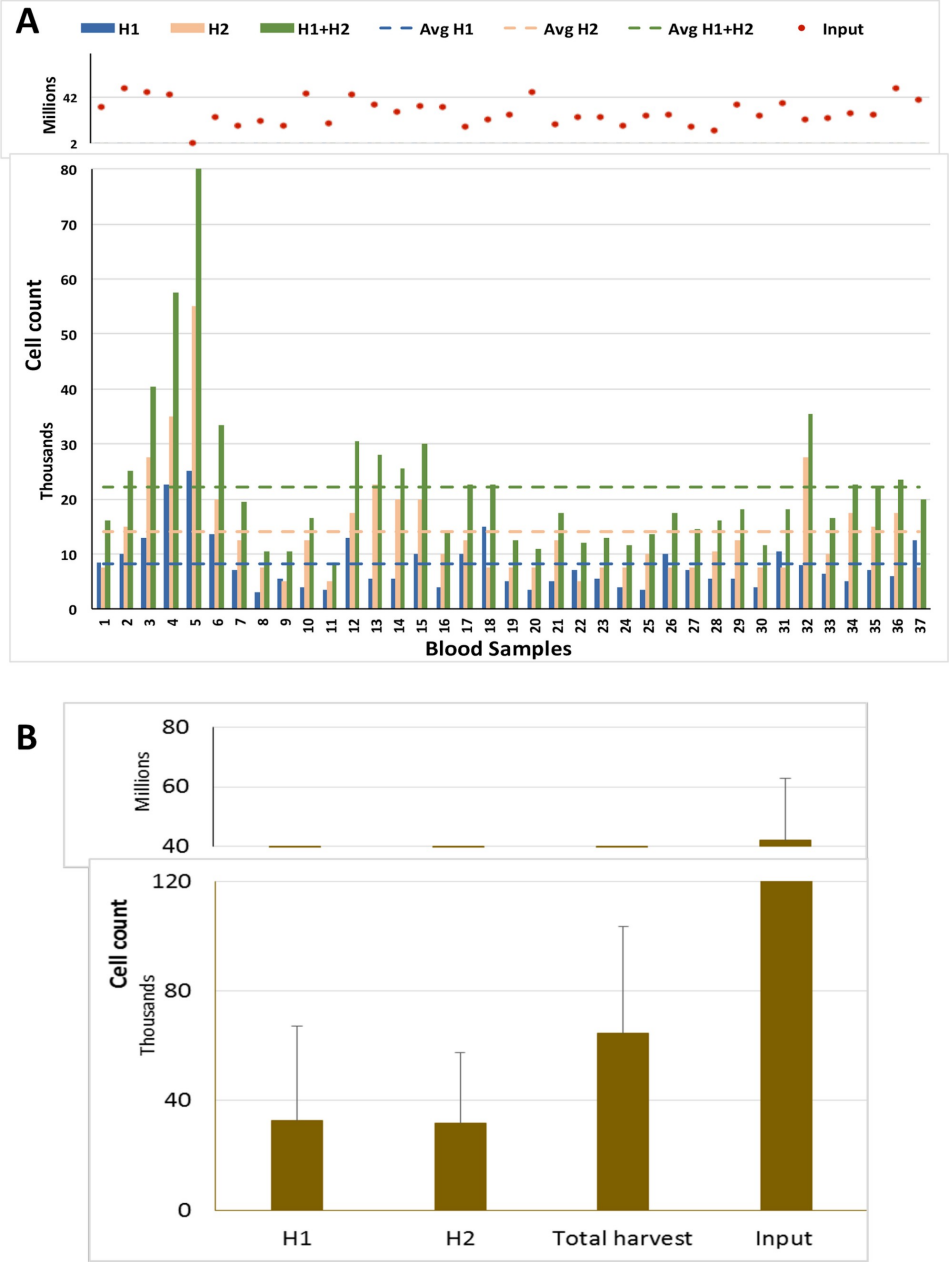
Yield of nucleated cells in Blood samples from metastatic patients using Parsortix system.

Interestingly, as a group, patients with metastatic estrogen receptor positive BC, yielded average harvested cell counts from blood that were significantly higher than that of non-metastatic patients, (8,100, range 3,500–25,000 versus 5,270 respectively, p = .005) (**Figs 3** and **4**), although there was no difference in the average number of input cells (29 million, range 17–49 million), and no obvious differences in collection methods, or other pre-analytical variables.

Total nucleated cell input and count in harvest fractions of BM specimens (A) and average number of cells in each fractions (B).

### Capture and recovery of cancer cells

While reduction of background nucleated cells is an important determinant for enrichment efficiency, retention and recovery of target tumor cell populations is critical to evaluate overall platform performance. To analyze the efficiency of BC cell capture and recovery from BM specimens, fluorescently labelled SKBR3 cells were diluted into BM. After enrichment in the Parsortix cassette, the number of fluorescent cells collected in the H1 and H2 eluates, as well as the number of cells retained in the cassette after harvest were determined using six BM specimens (4 from BC patients and two from healthy volunteers) (**Table 1**). Percent capture of spiked cells in the cassette ranged from 30–84% with an average of 51%. Suggesting that 16–70% of cells were lost either during processing or flow through. The percentage of spiked cells recovered in the H1 eluate ranged from 4–61% with an average of 26%. On average, only an additional 4% of tumor cells were recovered with an additional elution (H2) while, as discussed above, the second elution contained predominantly background nucleated cells which reduced overall purity. Average enrichment of cancer cells, as a reflection of the number of cancer cells recovered and the reduction of WBC, was approximately 306 fold but varied widely from approximately 60 to 890. Combining H1 and H2 harvests to increase the number of cancer cells recovered greatly reduced the enrichment from 306- to 98-fold.

**Table 1:** Harvest cell counts from spiking experiments using labeled SKBR3 cells in BM.

	BM1	BM2	BM3	BM4	BM5	BM6
**Total nucleated BM cells (X 10**^**−7**^**)**	5.2	1.6	8.0	1.9	8.6	8.6
**Total cells spiked**	124	108	248	181	154	157
**H1 (%)**	76 (61)	28 (26)	84 (34)	7 (4)	13 (8)	35 (22)
**H2 (%)**	12 (10)	0 (0)	24 (10)	0 (0)	0	4(3)
**Cassette (%)**	16 (13)	10 (9)	80 (32)	59 (32)	33 (21)	32 (20)
**Total capture (%)**	104 (84)	38 (35)	188 (76)	66 (36)	46 (30)	71 (45)
**Fold Enrichment**	423	143	98	61	220	890

Percentage of cells to the total number of cells spiked is given in parenthesis.

### Detection of DTC associated biomarkers in enriched cell populations

**Fig 5:**
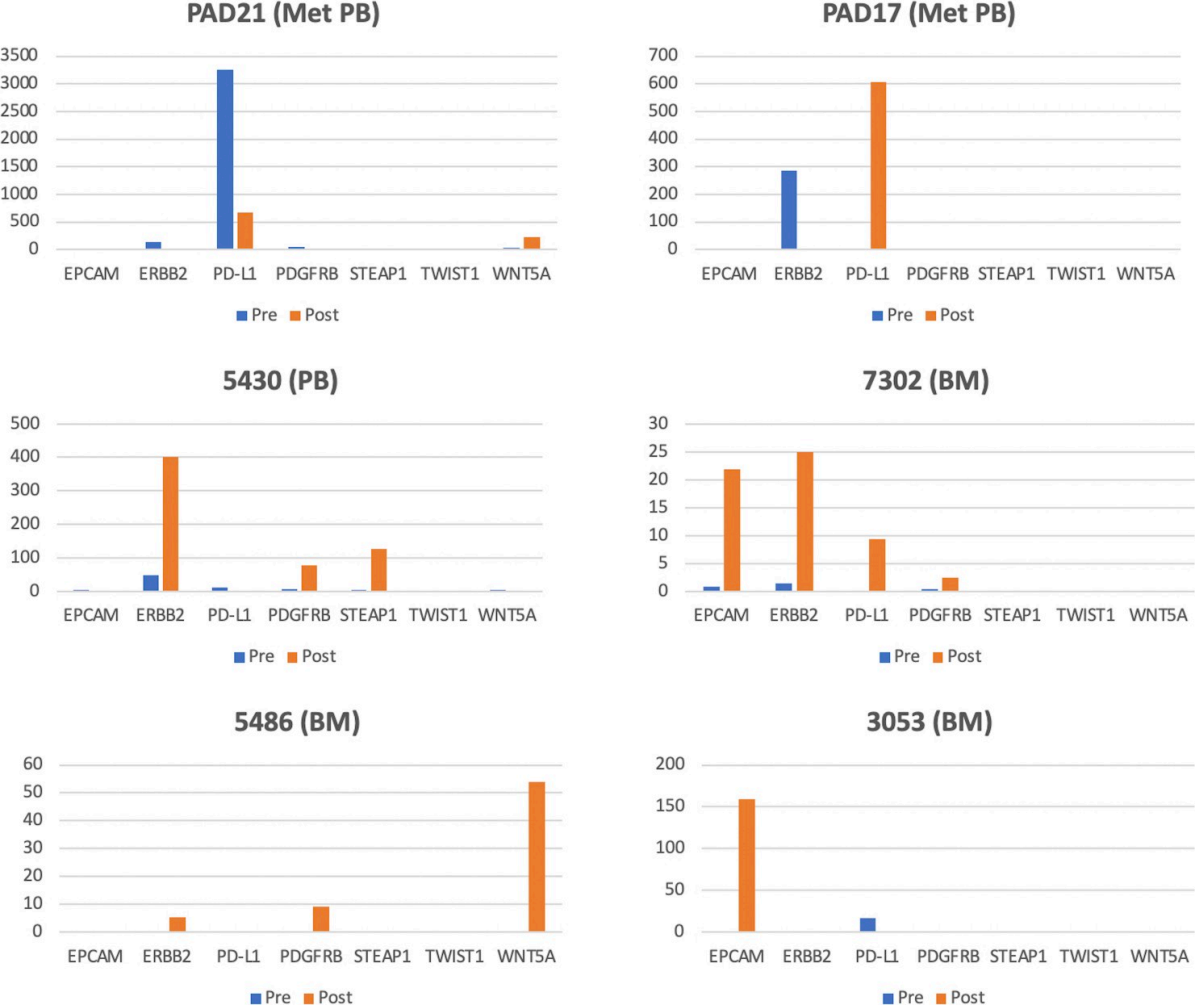
Gene expression of a 7-transcript panel in six different Bone Marrow (BM) and Peripheral Blood (PB) samples from metastatic (Met) and non-metastatic breast cancer patients. Individual gene expression values from cell samples pre- (blue) and post- (orange) Parsortix filtration are expressed as positive droplets per 100,000 GAPDH positive droplets.

Because of our on-going interest in detecting and classifying DTCs based upon their molecular (gene expression) signatures, we next evaluated whether the enrichment documented with tumor cells spiked into patient BM samples translated into more sensitive detection of native BM DTCs using molecular biomarkers. Accordingly, we assessed quantitative levels of gene expression for seven transcripts that have been previously associated with the presence of BM DTCs in BC patients [32]—*EPCAM*, *ERBB2*, *PD-L1*, *PDGFRB*, *STEAP1*, *TWIST1*, and *WNT5A*- by droplet digital PCR (**ddPCR**) pre- and post-Parsortix enrichment. 10 BM and 9 peripheral blood specimens were analyzed. Overall, 6 of 10 pre-enriched BM and 7 of 9 pre-enriched peripheral blood specimens had detectable levels of one or more of six transcripts (**Table 2**). Post-filtration (H1 eluate), 6 of 10 BM, however, only 3 of 9 peripheral blood samples had detectable levels of one or more transcripts. A pair-wise comparison of pre- and post-enrichment BM and peripheral blood revealed gene expression that was enhanced quantitatively by 2- to 25-fold or that was only detectable after filtration (**Fig 5**). However, expression of several marker genes was non-detectable or greatly diminished after filtration, both in peripheral blood and BM, suggesting that DTCs or other rare cell populations from which these genes were expressed were lost during the filtration process. Insufficient follow up time did not allow for correlation of quantitative gene expression in BM or peripheral blood with disease progression, but there was no obvious correlation between stage (metastatic vs. non-metastatic) and the number of positive markers or the quantitative level of individual transcript expression.

**Table 2:** Gene expression in blood or BM pre- and post-filtration based on expression of one or more gene marker transcripts.

Patient / Sample	Number	Positive Pre-filtration	Positive Post-filtration
**Non-Met BM**	10	6	6
**Non-Met Blood**	4	4	1
**Met Blood**	5	3	2

### Single cell RNA sequence analysis of enriched cells

**Fig 6:**
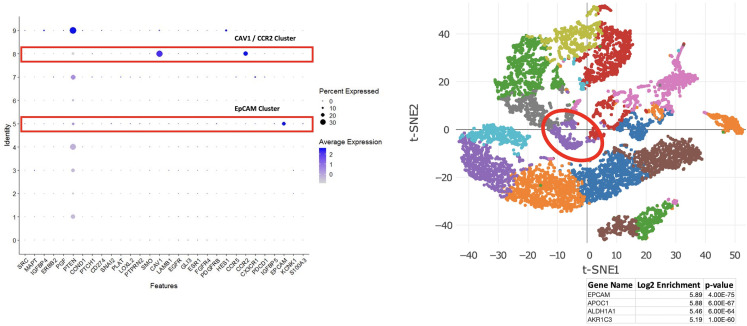
**(*Left*) Expression of DTC gene panel across cell clusters from TNBC BM sample.** (*Right*) tSNE plot highlighting the EPCAM-specific cluster (2% of the cell population) and list of most significant, differentially expressed transcripts in that cluster.

To evaluate the use of enriched cell populations to perform single cell gene expression profiling (RNA sequencing), H1 eluates from three BM samples obtained from treatment naïve, triple negative BC patients (approximately ~15,000 enriched cells from each) were used for 10X genomics library preparation and RNA sequencing. **Fig 6** shows the results for a representative sample. Unsupervised cluster analysis of ~9,500 cell libraries revealed two distinct clusters of cells that were enriched for two marker transcripts which we and others have previously associated with DTC-positive BM from BC patients: *CAV1* and *EpCAM* [32]. In whole BM gene expression analyses, expression of ***CAV1*** is frequently associated with early distant disease relapse [32]. In fact, this patient experienced rapid disease progression 9 months after diagnosis. An independent cluster of cells enriched for *EpCAM* expression, a well-recognized marker of DTCs [33], represented ~ 2% of the total enriched cell population. Assuming an average population of 10 DTCs per million BM cells [33], this also suggests that the Parsortix platform was able to perform at least a 500-fold enrichment of DTCs from BM. Moreover, the cluster of EpCAM enriched cells were also enriched for several other genes of clinical interest including *APOC1*, a biomarker of tumor progression [34], *ALDH1A1*, a marker of tumor cell “stemness” [35], and *AKR1C3*, a marker of doxorubicin resistance [36]. Thus, viable cell enrichment with the microfluidic platform not only allowed detection of DTC populations but as shown by this example, allowed for the delineation of independent DTC populations (EpCAM positive and EpCAM negative) whose molecular phenotypes may have distinct, additive, or synergistic consequences for treatment and monitoring of DTC populations to mitigate metastatic progression.

## Discussion

After definitive surgical resection of the primary tumor, DTCs remain a source of distant metastatic disease progression, even in early stage BC patients [37–39]. Thus, isolation and molecular characterization of DTCs for targeted therapy to prevent overt metastases is of high clinical significance. Multiple methods have been used to enrich for DTCs. Due to the molecular heterogeneity of DTCs, enrichment methods based on physical properties rather than specific cell surface epitopes are likely to capture a broader population of DTCs [23]. In this study we tested the efficacy of the Parsortix microfluidics system, which uses selective filtration, to enrich for DTCs based on deformability and cell size, the latter of which is generally greater for tumor cells than most peripheral blood cells [26]. There are several advantages of this system including ease of use, epitope independence, and the retrieval of viable cells. This system has been used and, until now, optimized to enrich for CTCs.

To date, the most common CTC enrichment methodology is based on retrieval of cells expressing epithelial surface markers such as EpCAM. However, a large percentage of CTCs, as well as DTCs, may have reduced expression of EpCAM especially as they undergo epithelial to mesenchymal transition [40, 41], resulting in a EpCAM low or negative CTCs [40, 42]. In direct comparison of the two methodologies, the Parsortix system has been reported to outperform EpCAM based capture for non-small cell [43] and prostate CTCs [24].

In reported studies, capture rates of CTCs from blood ranged from 28 to 92% [24, 25, 28, 44] and harvest rates ranging from 20% to 80 percent [24] (**S3 Table**). For the BC cell line SKBR3 diluted into BM, we found cell capture within the cassette ranged from 30–84% which is consistent with the results for capture of CTCs. In addition, we found that greater than half of the spiked captured cells could be eluted off the cassette in 66% of the trials. (**Table 1**). This is consistent reported results of 20 to 87% recovery of CTCs from blood.

Because of the cellular complexity of BM compared with blood, techniques that enrich for CTCs may work with reduced efficiency when trying to capture DTCs. Using the Parsortix system, we found that the number of contaminating WBC using blood from early stage breast cancer patients was similar to that reported in the literature (**S3 Table**). However, despite similar starting cell numbers, contaminating WBC harvested using BM in the Parsortix system about twice as high as blood. Importantly, the recovery of spiked cancer cells from BM was not affected, since the percentage recovered from BM was in a similar range as that recovered from blood (**S3 Table**).

Interesting, we found a significantly higher number (50% higher) of nucleated cells recovered using blood collected from patients with metastatic estrogen receptor positive BC compared to blood samples from non-metastatic patients (**S3 Table**) though similar numbers of WBC were input into the system. It is unlikely that this increase is solely due to an increase in the number of CTCs since CTC levels are generally on the order of a few 100 per 7.5 ml blood in patients with metastatic disease [45]. It is possible that the increased yield of nucleated cells are due to CTC clusters or CTC-WBC clusters which have been implicated in metastatic disease [46, 47] and may disrupt the normal flow of peripheral cells through the device.

Cells retrieved from the Parsortix system are viable and thus can be used for multiple downstream analyses including tissue culture, single cell sequencing, PCR, and IHC [24, 43]. We assessed the expression of CTC/DTC associated gene expression by ddPCR. As expected, no gene expression was detected in 45% of the BM and 66% of the blood samples. However, in 36% of the BM, and 33% of blood enrichment as assessed by gene expression analysis was successfully achieved for 35 percent of the samples (4 DTC, 3 CTCs). In 5% of the samples, there was a likely loss of cells since gene expression detected in the pre-filtered samples was not detected in the harvested cells. While this may be due to technical issues, every effort was made to run all samples with uniform processing. More likely explanations are that expression loss was due to the properties of the cells which either caused them to be retained in the cassette or to flow through or that expression of genes from contaminating cells may interfere with expression of genes relevant to BC [44] or that some of the gene expression is emanating from cell types other than CTCs/DTCs which are differentially enriched by Parsortix.

We found that enriched DTCs were sufficient in number and viability to serve as a template for single cell RNA sequencing analysis and biologically and clinically relevant BC expression profiles were detected in one or possibly more subpopulations of isolated cells. To date, single cell sequencing has been performed on DTCs isolated using EpCAM enrichment [48, 49]. The role of EpCAM low or negative CTCs in the metastatic process is under investigation [41, 50]. Now it will be possible to molecularly interrogate EpCAM negative or low DTCs using this system.

We report that the Parsortix system, an automated size and flow-based CTC enrichment platform can be successfully used to enrich DTCs from BM of BC patients with minor modifications. We demonstrate recovery, enrichment, and downstream molecular analysis similar to that achieved with blood. The Parsortix system has several advantages over current methodologies including the relative ease of operation, minimal handling of specimen and recovery of viable cells.

## Supporting information

S1 Fig Reduction of nucleated cells from blood and BM samples after processing through Parsortix system. (TIFF)

S1 Table Details of probes used for gene specific amplification using DDPCR. (PDF)

S2 Table Cassette optimization for processing BM specimen. (PDF)

S3 Table Literature review of captured cell yield using Parsortix. (PDF)
