# Supplementary figures and images for: A microfluidic-based filtration system to enrich for bone marrow disseminated tumor cells from breast cancer patients
Source: PLoS One. 2021 May 14;16(5):e0246139. doi: 10.1371/journal.pone.0246139 (PMC8121342; doi:10.1371/journal.pone.0246139)

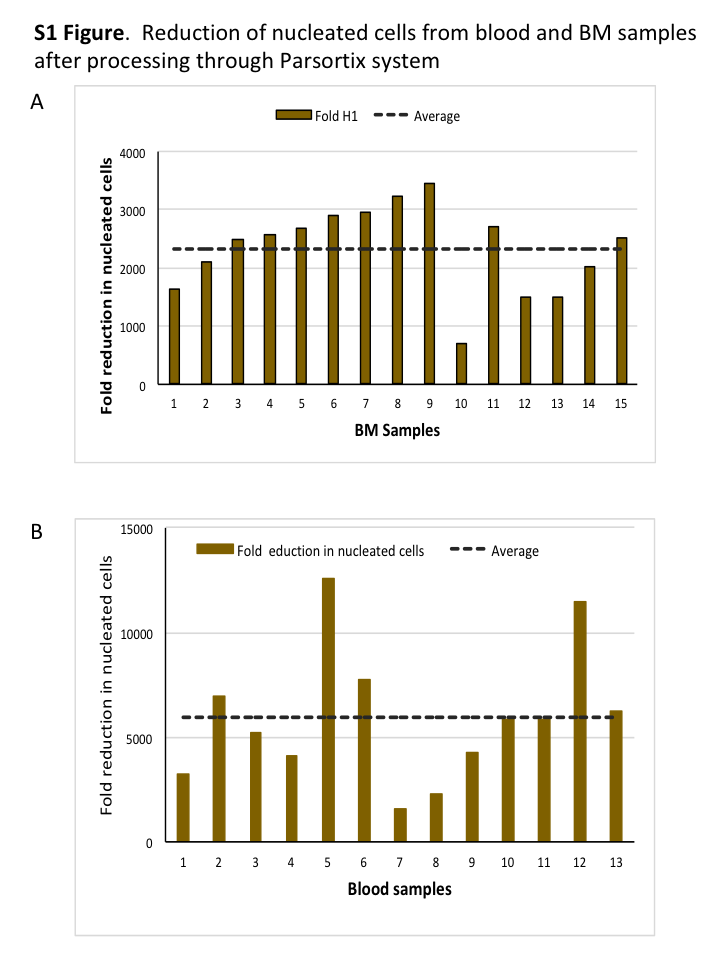

Supplement: S1 Fig — (TIFF) [file pone.0246139.s001.tiff]
