# Supplementary material for: A microfluidic-based filtration system to enrich for bone marrow disseminated tumor cells from breast cancer patients
Source: PLoS One. 2021 May 14;16(5):e0246139. doi: 10.1371/journal.pone.0246139 (PMC8121342; doi:10.1371/journal.pone.0246139)
Supplement: S1 Table — (PDF) [file pone.0246139.s002.pdf]

S1 Table: Details of probes used for gene specific amplification using DDPCR

Transcript	TaqMan® Gene Expression Assay ID
EPCAM	Hs00901885_m1
ERBB2	Hs01001580_m1
PDL-1	Hs00204257_m1
PDGFRB	Hs01019589_m1
STEAP1	Hs00185180_m1
TWIST1	Hs00361186_m1
WNT5A	Hs00998537_m1
