# Supplementary material for: A microfluidic-based filtration system to enrich for bone marrow disseminated tumor cells from breast cancer patients
Source: PLoS One. 2021 May 14;16(5):e0246139. doi: 10.1371/journal.pone.0246139 (PMC8121342; doi:10.1371/journal.pone.0246139)
Supplement: S2 Table — (PDF) [file pone.0246139.s003.pdf]

S2 Table: Cassette optimization for processing BM specimen

				BM1	BM2	BM3	BM4	BM5	BM6			
**Pt ID**	**8219**	**1179**	**8219**	**2595**	**1184**	**1185**	**3053-2**	**7370-1**	**7370-2**	**9391**	**8219**	**8167**
**Cassette gap (um)**	4.5	6.5	6.5	6.5	6.5	6.5	6.5	6.5	6.5	8	8	10
**Total cell input ($x10^{-6}$)**	66.6	60.6	66.6	52	16	80	19	86	86	19.5	66.6	-
**BC cells spiked**	203	~200	158	124	108	248	181	154	157	~200	178	124
**BC captured (%)**	47 (23)	133 (66)	152 (96)	104 (84)	38 (35)	188 (76)	66 (36)	46 (30)	71 (45)	136	178 (100)	52 (42)
**BC harvested (%)**	20 (10)	69 (35)	17 (11)	88 (71)	28 (26)	108 (44)	7 (4)	13 (8)	39 (25)	109 (55)	34 (20)	10 (8)
**BC retained in cassette (%)**	27 (13)	64 (32)	135 (85)	16 (13)	10 (9)	80 (32)	59 (32)	33 (21)	32 (20)	27 (13.5)	144 (81)	42 (34)
**Total harvest cell count ($x10^{-4}$)**	11.3	2.8	21	75.5	29	28	12	32	21.5	14.6	93	37.2
**Fold enrichment (FirstHarvest**	589	2133	317	423	143	98	61	220	89	133	71	
