# Supplementary material for: A microfluidic-based filtration system to enrich for bone marrow disseminated tumor cells from breast cancer patients
Source: PLoS One. 2021 May 14;16(5):e0246139. doi: 10.1371/journal.pone.0246139 (PMC8121342; doi:10.1371/journal.pone.0246139)
Supplement: S3 Table — (PDF) [file pone.0246139.s004.pdf]

S3 Table : Literature review of captured cell yield using Parsortix
*number of leukocytes per ml blood=10^6

**Author**	**Cancer cell line**	**Volume loaded**	**Cell capture in cassette** (%)	**Cell Harvested from cassette** (% of captured cells)	**Recovery** (capture cells X harvested cells)	**Harvested nucleated cells** (number total)	**Fold reduction in WBC***
Pillai	Breast cancer	2 ml **BM**	30-84%	11-73%	4-61%	11300	2550
PIllai	Breast cancer	7 ml blood early stage				5270	5950
Pillai	Breast cancer	7 ml blood metastatic				8100	4570
Hvichia[25]	Multiple	4 ml blood	42-70%	54-69%	22-49%	200-5000	800-20,000
Maertens[27]	Renal cancer	5 ml blood			30-87%		
Porras[44]	Breast cancer	7.5 ml blood	61-75%		20%	542-5174	
Miller[26]	Breast cancer	5 ml blood	66-92%	62-84%	38-77%	1000-4000	
Lampignano[40]	Breast cancer	7.5 ml blood	46-80%	42-100%	20-80%		
Xu[24]	Prostate cancer	3 ml blood	58-66%	35-50%	20-52%	927	
Chudziak[28]	Colorectal cancer	4 ml blood			57-83	2610-3069	
Franken[50]	Breast cancer	7.5 ml blood	56-78%	55-75%	42%		
Obermayr[51]	Small cell lung cancer	10 ml blood	28%				
